# All Blood Brain Barrier Cell Types Demonstrate Capability to Influence Differential Tenofovir and Emtricitabine Metabolism and Transport in the Brain

**DOI:** 10.1021/acsptsci.4c00510

**Published:** 2024-10-18

**Authors:** Hannah N. Wilkins, Stephen A. Knerler, Ahmed Warshanna, Rodnie Colón Ortiz, Kate Haas, Benjamin C. Orsburn, Dionna W. Williams

**Affiliations:** † Department of Pharmacology and Molecular Sciences, Johns Hopkins University School of Medicine, Baltimore, Maryland 21205, United States; ‡ Department of Pharmacology and Chemical Biology, Emory University School of Medicine, Atlanta, Georgia 30322, United States; § Department of Neuroscience, Johns Hopkins University School of Medicine, Baltimore, Maryland 21205, United States; ∥ Department of Molecular and Comparative Pathobiology, Johns Hopkins University School of Medicine, Baltimore, Maryland 21205, United States

**Keywords:** blood brain barrier, antiretroviral therapy, central nervous system, drug disposition, drug transporter, drug metabolism

## Abstract

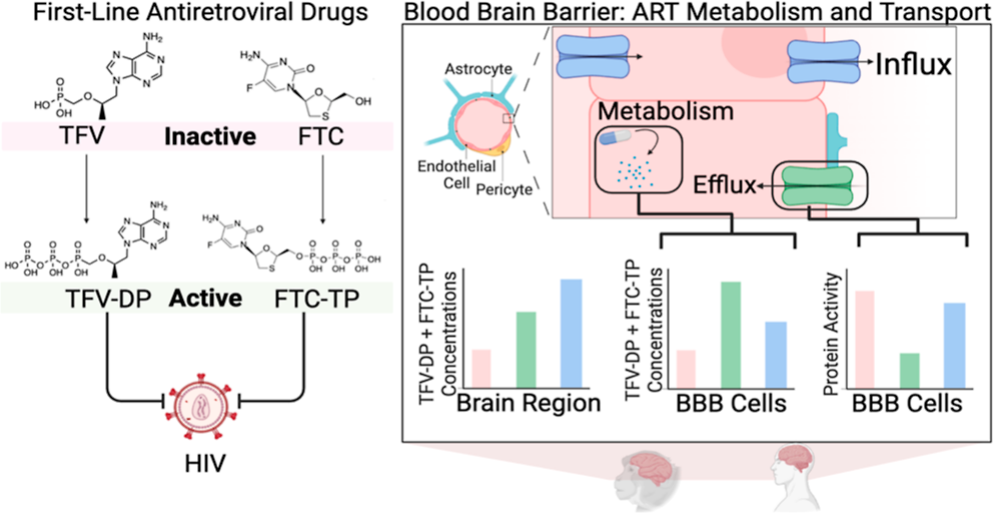

The blood brain barrier (BBB) represents a significant obstacle in brain drug penetration that challenges efforts in the treatment of neurological disorders. Therapeutically targeting the brain requires interactions with each BBB cell type, including endothelial cells, pericytes, and astrocytes. Yet, the relative contribution of these BBB cell types to the mechanisms that facilitate brain drug disposition is not well characterized. Here, we use first-line antiretroviral therapies, tenofovir (TFV) and emtricitabine (FTC), as models to investigate the mechanisms of drug transport and metabolism at the BBB that may influence access of the drug to the brain. We evaluated regional and cell-type-specific drug metabolism and transport mechanisms using rhesus macaques and in vitro treatment of primary human cells. We report heterogeneous distribution of TFV, FTC, and their active metabolites, which cerebrospinal fluid measures could not reflect. We found that all BBB cell types possessed functional drug-metabolizing enzymes and transporters that promoted TFV and FTC uptake and pharmacologic activation. Pericytes and astrocytes emerged as pharmacologically dynamic cells that rival hepatocytes and were uniquely susceptible to modulation by disease and treatment. Together, our findings demonstrate the importance of considering the BBB as a unique pharmacologic entity rather than viewing it as an extension of the liver, as each cell type possesses distinct drug metabolism and transport capacities that contribute to differential brain drug disposition. Further, our work highlights pharmacologically active pathways at the BBB that may regulate brain drug disposition and impact therapeutic efforts to alleviate neurologic disease.

## Introduction

The global impact of neurologic disease is astounding, as it affects every developmental stage and virtually all facets of life, including movement, cognition, and the capacity for human connection. For these reasons, substantial drug discovery efforts focus on developing small-molecule therapeutics whose targets have implications in the prevention and treatment of neurologic disease. Yet, even when putative therapies with great clinical promise are identified, the blood brain barrier (BBB) creates a major obstacle for central nervous system (CNS) penetration that poses significant challenges in achieving pharmaceutical efficacy.^1^ As such, understanding the mechanisms underlying drug distribution in the brain is poised to have a wide-reaching impact on millions of lives impacted by neurologic disease.

While endothelial cells encompass the primary portion of the brain microvasculature, the essentiality of the surrounding neurovascular unit in maintaining appropriate BBB function cannot be ignored. Emerging evidence demonstrates that pericytes and astrocytes are more than support cells and play distinct roles from endothelial cells, without which appropriate BBB function would not occur.^2,3^ Even still, understanding of pericyte and astrocytic contributions to the BBB is primarily restricted to permeability and integrity. As such, their capacity to contribute to CNS drug disposition has not been widely considered. This limitation becomes even more apparent due to the regional variability that exists at the BBB wherein diverse anatomic sites have differential pericyte and astrocyte innervation that is dynamically regulated.^4^ The relative contribution of endothelial cells, pericytes, and astrocytes to regional heterogeneity in CNS drug distribution remains unknown, the understanding of which will be important to identifying mechanisms to target drug-specific anatomic sites in the brain.

We aimed to address this question by evaluating drug metabolizing enzymes and membrane-associated transporters, as they are essential for drug efficacy, biotransformation, elimination, and distribution in tissues. These pharmacological pathways of drug metabolism and transport are primarily characterized in peripheral organs, including the liver, yet remain largely unknown at the BBB. Our study used antiretroviral therapies (ART) primarily used for the prevention and treatment of human immunodeficiency virus-1 (HIV) to probe this question, as they are uniquely positioned to serve as an investigative model for CNS drug disposition. The CNS represents one of the most challenging viral sanctuaries for HIV as it invades the brain within two weeks of infection, resulting in significant neurologic consequences in ∼50% of people living with the virus.^5,6^

Here, we used the first-line nucleot(s)ide reverse transcriptase inhibitors, tenofovir (TFV) and emtricitabine (FTC), to (1) evaluate regional differences in drug distribution across the brain and (2) elucidate differential drug transport and metabolizing capabilities of brain endothelial cells, pericytes, and astrocytes, which may serve as a model of CNS drug disposition pathways that determine drug access at the dynamic microenvironment of the BBB. With a combination of in vivo studies in simian immunodeficiency virus (SIV)-infected and ART-treated rhesus macaques and in vitro HIV exposure and ART treatments of cell monocultures, we evaluated the capacity of each cellular component of the BBB to uptake and convert TFV and FTC into their pharmacologically active metabolites using a combination of liquid chromatography/mass spectrometry (LC–MS/MS), proteomics by mass spectrometry, and drug transporter and metabolizing enzyme functional assays. Our findings demonstrate that all cells at the BBB possess drug metabolizing and transporting capabilities that can contribute to CNS drug bioavailability and efficacy, which may inform pharmacodynamics, drug targeting, and drug discovery. Further, our work indicates that the BBB is a dynamic, pharmacologically active microenvironment that responds differentially to disease and CNS small-molecule therapeutics in a cell-dependent manner.

## Results and Discussion

### TFV and FTC Are Heterogeneously Distributed among Brain Regions and BBB Cell Types

**Figure 1:**
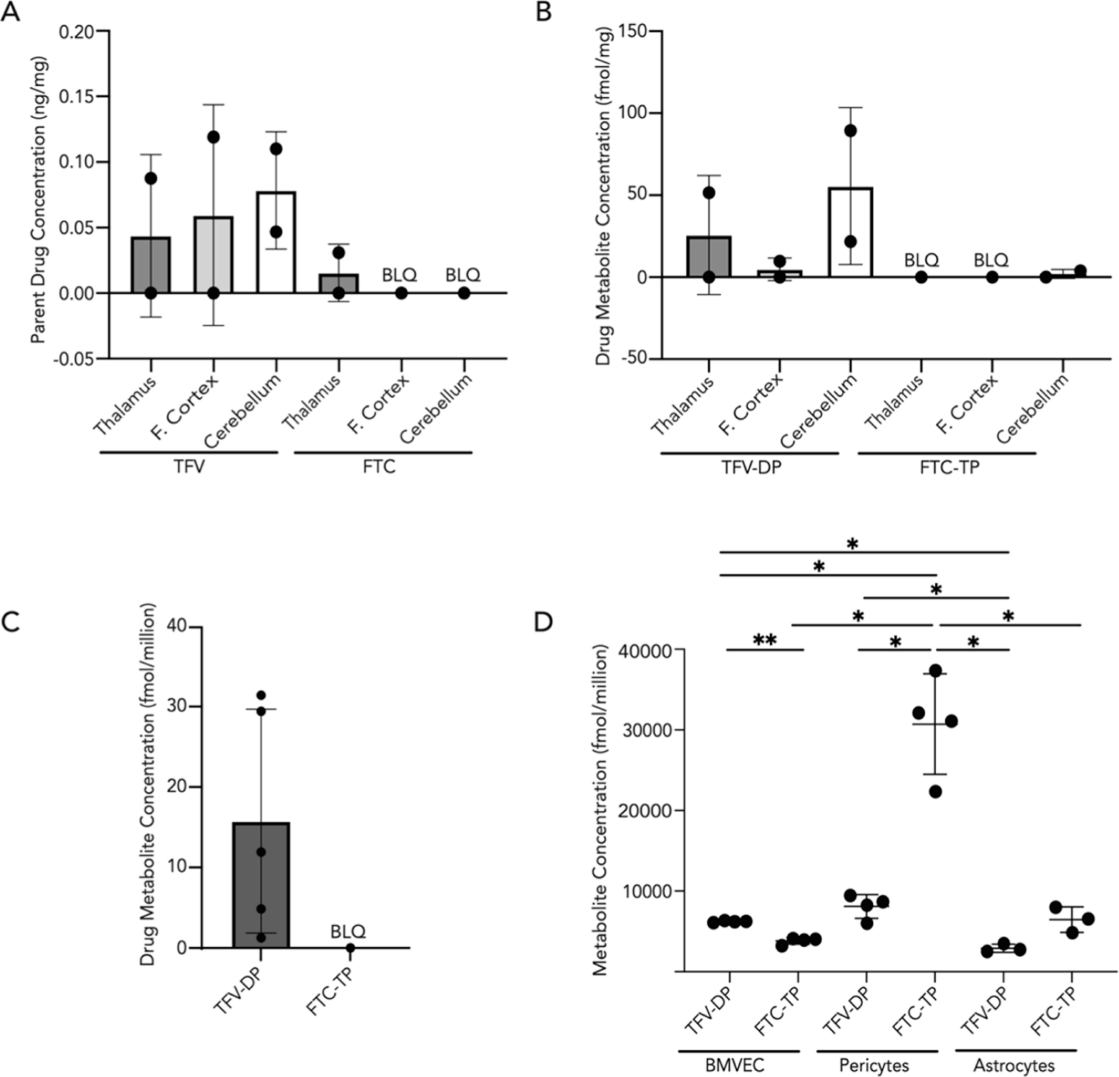
TFV and FTC parent and metabolite quantification in BBB cells and rhesus macaque tissues. (A,B) The thalamus, frontal cortex (denoted as F. Cortex), and cerebellum were sectioned from rhesus macaque brains collected 200 days post inoculation with SIVmac251 followed by a subcutaneous dose of 20 mg/kg TFV, 40 mg/kg FTC, and 2.5 mg/kg DTG administered once daily. Within these brain tissues, (A) parent TFV and FTC as well as (B) TFV-DP and FTC-TP metabolites were quantified by LC–MS/MS from the cerebellum, frontal cortex, and thalamus (*n* = 2 macaques, represented as individual dots). (C) Astrocytes were obtained from healthy rhesus macaques that received six months of a once daily intramuscular ART administration (5.1 mg/kg TFV, 50 mg/kg FTC, and 2.5 mg/kg DTG). Astrocytes were isolated from five macaque brains (represented as individual dots), and TFV-DP and FTC-TP were quantified by LC–MS/MS. Below limit of quantification (BLQ) samples reported (represented as a singular dot) had concentrations less than 5 fmol/sample (TFV-DP), 50 fmol/sample (FTC-TP), 0.05 ng/sample (TFV), and 0.25 ng/sample (FTC). (D) Primary human BBB cells were treated with 18 μM TFV, 2 μM FTC, and 6 μM DTG for 24 h at 37 °C, 5% CO_2_. After this time, TFV-DP and FTC-TP were quantified by LC–MS/MS. Three to four independent experiments were performed (represented as individual dots). Data represented as mean ± standard deviation. Statistical analysis was performed by 10.1.1 GraphPad Software, Inc., San Diego, CA, using Brown-Forsythe and Welch ANOVA. *p < 0.05, **p < 0.01, ***p < 0.001, and ****p < 0.0001.

It is well accepted that ART penetrates the brain to a lesser extent than peripheral tissues. Previously, ART cerebrospinal fluid (CSF) measurements were used as surrogates for brain concentrations.^7^ However, measurements within the brain demonstrated a lack of concordance with CSF concentrations.^8,9^ These studies indicate that first-line ART drugs, TFV and FTC, are CNS penetrant; however, the mechanisms at the BBB that facilitate their entry into the brain and contribute to heterogeneous CNS distribution remain poorly understood.^8−10^ To address this, we obtained brain from SIV-infected rhesus macaques that received a, once daily, 6-month ART regimen that included TFV and FTC and performed LC–MS/MS. We focused on thalamus, frontal cortex, and cerebellum as HIV infection of these brain regions is well characterized and the resultant neurologic deficits that occur represent an important public health consideration.^11,12^ We found that TFV concentrations were higher in all three tissues relative to FTC, which was below the limit of quantification for all brain regions except the thalamus (Figure 1A). Interestingly, TFV was heterogeneously present in the brain, wherein it was 78% higher in the cerebellum (0.0784 ± 0.0448 ng/mg) relative to the thalamus (0.0438 ± 0.0619 ng/mg) and 32% higher than the frontal cortex (0.0595 ± 0.0842 ng/mg).

We next evaluated the active metabolites of TFV and FTC, TFV-diphosphate (TFV-DP), and FTC-triphosphate (FTC-TP), respectively, by LC–MS/MS, as these are the only versions of these drugs capable of suppressing HIV (Figure 1B). TFV-DP concentrations were 116% and 1134% higher in the cerebellum (55.60 ± 47.80 fmol/mg) relative to the thalamus (25.75 ± 36.42 fmol/mg) and the frontal cortex (4.90 ± 6.92 fmol/mg), respectively, suggesting that a differential capacity exists for pharmacologic activation across brain regions (Figure 1B). Further, TFV-DP (55.60 ± 47.80 fmol/mg) was 2866% higher than FTC-TP (1.94 ± 2.74 fmol/mg) in the cerebellum, highlighting a differential accumulation of ART-active metabolites within brain regions. Similar to that which occurred for its parent drug, FTC-TP was below the limit of quantification for all brain regions except the cerebellum. We did not detect TFV, FTC, or their active metabolites in the brain obtained from untreated control rhesus macaques (data not shown). Additionally, the variability in TFV and FTC parent and metabolite measurements may be attributed to the interindividual differences in antiretroviral drug concentrations that have been previously observed between both rhesus macaques and humans.^13−15^ Variability in therapeutic measurements may also arise due to the interindividual differences reported in SIV viral load, disease progression, and virus, particularly for the SIVmac251 strain used in this study.^13,15^ Together, our findings demonstrate that, overall, TFV had greater CNS access than FTC. This is striking, as these therapies belong to the same ART class and have similar molecular profiles, including charge and molecular weight. Of importance, these in vivo findings confirm our previous work using an in vitro model of the human BBB indicating the relevance of this model as a tool in evaluating CNS ART disposition.^16^ Our current work confirms that variable TFV and FTC concentrations occurred across brain regions, consistent with evidence suggesting heterogeneous ART distribution in the brain,^8,9,17,18^ though this is an emerging area of investigation. Specifically, our findings are consistent with the ART heterogeneity observed in human and mouse brains but are distinct from other rhesus macaque studies,^7,14^ which may be attributed to differences in study design, including viral strain, ART regimen, and dosing duration. Further, we showed for the first time that this regional variability extended to TFV and FTC active metabolites. We identified the highest TFV and TFV-DP concentrations in the cerebellum, which is vulnerable to HIV infection and damage.^19^ Historically, CSF measurements used to predict CNS ART concentrations suggested increased FTC penetrance in the brain relative to TFV, which our findings do not support.^20,21^ Rather, our results further highlight the disparity between CSF and brain tissue ART measurements and caution against its use as a proxy for parenchymal concentrations.^20,21^ These results continue to elucidate the differential distribution of TFV and FTC within the CNS and, more importantly, the heterogeneous measurement of their pharmacologically active metabolites across brain regions.

TFV and FTC entry into the brain requires important interactions with the BBB. It remains unclear if, during these interactions, ART is retained within the cells comprising the BBB, or if instead they are transported in their entirety into the brain parenchyma. This is particularly important as astrocytes and pericytes are permissive to HIV and infection dysregulates their essential contributions to BBB function.^22,23^ While astrocyte uptake of TFV and FTC is known, their intracellular accumulation of TFV-DP and FTC-TP, and thus their antiretroviral therapeutic potential, has not been evaluated previously.^24,25^ To address this, we quantified TFV-DP and FTC-TP in astrocytes isolated from the brain tissue of uninfected rhesus macaques that received a, once daily, 6-month ART regimen that included TFV and FTC (Figure 1C). We determined that TFV-DP was quantifiable in astrocytes (15.76 ± 13.92 fmol/million) and that concentrations were similar to reported EC90 for TFV measured in other cell types from rhesus macaques.^26^ In contrast and consistent with our findings from the rhesus macaque brain tissue, FTC-TP was below the limit of quantification in astrocytes (Figure 1C). No sex-based differences occurred for astrocyte TFV and FTC quantification (data not shown). We performed similar analyses on brain capillaries, containing brain microvascular endothelial cells and pericytes, from these same macaques. However, cell yields were too low to facilitate detectable quantification (data not shown). Nevertheless, our rhesus macaque measurements may indicate ART therapeutic potential in astrocytes, which is essential to viral eradication of the brain as these cells are infected by HIV and contribute to a persistent CNS reservoir.^27,28^

We next performed in vitro TFV and FTC treatment for all three cells comprising the BBB as we were unable to perform this comparative analysis with rhesus macaques. Prior studies observed that TFV and FTC accumulate intracellularly within endothelial cells, pericytes, and astrocytes that result in differential cell-type-specific concentrations.^24,25^ Although these findings suggest that BBB cells may have distinct capabilities in accumulating TFV and FTC, prior studies did not evaluate whether these cells were capable of TFV and FTC metabolism. To determine whether BBB cells are capable of parent drug metabolism into TFV-DP and FTC-TP, monocultures of primary human astrocytes, endothelial cells, and pericytes were exposed to a regimen composed of rhesus macaque equivalent ART doses for 24 h, after which time the cells were lysed, and TFV-DP and FTC-TP concentrations were determined by LC–MS/MS. In accordance with our previous findings, TFV and FTC did not impact cell viability (data not shown).^16^ Importantly, all BBB cells expressed cell-type-enriched markers present in vivo, including VE-cadherin, Alanyl Aminopeptidase (ANPEP), and Glial fibrillary acidic protein (GFAP) for endothelial cells, pericytes, and astrocytes, respectively, confirming cell identity (Figure S1). All BBB cell types demonstrated the capability of metabolizing TFV and FTC into their active metabolites (Figure 1D). However, significant differential concentrations of TFV-DP and FTC-TP occurred among the cells. TFV-DP was 113% (*p* = 0.0337) and 178% (*p* = 0.0220) higher in endothelial cells (6208 ± 109.50 fmol/million) and pericytes (8092 ± 1482 fmol/million), respectively, as compared to astrocytes (2913 ± 493.50 fmol/million) (Figure 1D). Similar differential concentrations occurred for FTC-TP, where pericytes (30,723 ± 6220 fmol/million) possessed 804% and 476% higher intracellular levels as compared to endothelial cells (3820 ± 409.60 fmol/million, *p* = 0.0203) and astrocytes (6447 ± 1587 fmol/million, *p* = 0.0133), respectively (Figure 1D). We also determined that some of the BBB cells exhibited a preferential capacity to metabolize ART. Endothelial cells had 62% higher TFV-DP (6208 ± 109.50 fmol/million) concentrations compared to FTC-TP (3820 ± 409.60 fmol/million, *p* = 0.0094), while the converse occurred in pericytes that had 379% higher FTC-TP (30,723 ± 6220 fmol/million) concentrations than TFV-DP (8092 ± 1482 fmol/million, *p* = 0.0356) (Figure 1D). Conversely, there was no statistical difference in the intracellular concentrations of TFV and FTC active metabolites in astrocytes (Figure 1D). Interestingly, we did not detect FTC-TP in rhesus macaque astrocytes and were surprised to quantify FTC-TP in human astrocytes in vitro. This finding suggests that astrocytes possess the capacity to pharmacologically transform FTC into its active metabolite should efficacious doses be achieved in the brain. While the metabolism of TFV and FTC in BBB cells initially appears promising for treating the brain during HIV, our findings raise concern for the utility of nucleotide analogues as putative CNS therapies. Our work suggests that local drug metabolism in endothelial cells, pericytes, and astrocytes may limit drug availability in the brain parenchyma by restricting it to cells at the BBB, as to our knowledge, there are no known transporters for the negatively charged, high molecular weight, and bulky TFV and FTC metabolites—suggesting that they may be retained within these cells rather than being released into the brain parenchyma.

### BBB Cells Differentially Express TFV and FTC Transporters and Nucleotide-Metabolizing Enzymes

**Figure 2:**
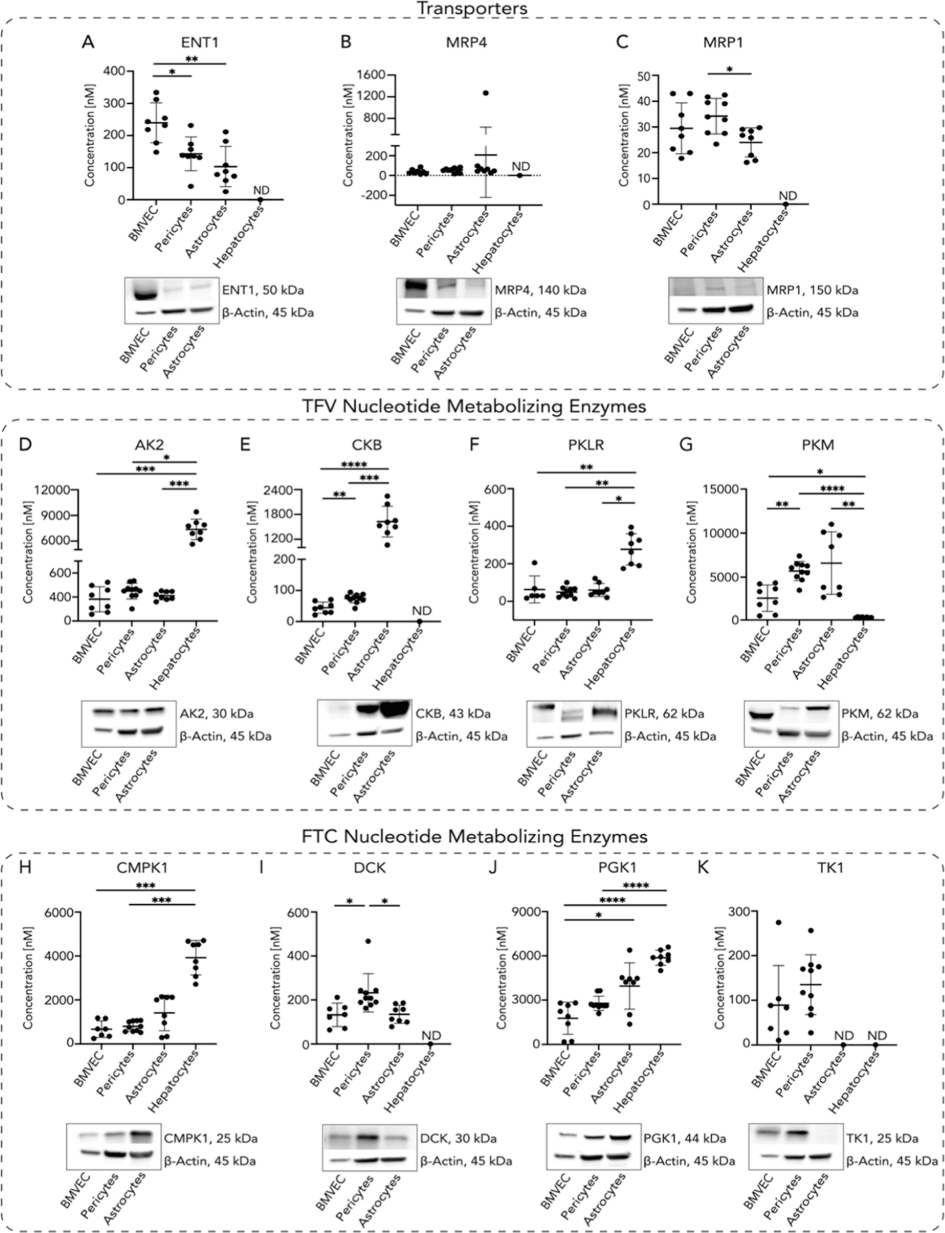
BBB cells differentially express TFV and FTC transporters and nucleotide-metabolizing kinases. Primary human BBB cell monoculture and hepatocyte lysates were processed for LC–MS/MS-based proteomics and protein concentrations for (A) ENT1, (B) MRP4, and (C) MRP1 TFV/FTC transporters, (D) AK2, (E) CKB, (F) PKLR, and (G) PKM TFV nucleotide-metabolizing kinases, as well as (H) CMPK1, (I) DCK, (J) PGK1, and (K) TK1 FTC nucleotide-metabolizing kinases, each with respective protein Western blots to confirm protein abundance in BBB cells. Concentrations of proteins of interest were measured by Proteomic Ruler. Four to five independent experiments with two LC–MS/MS injection replicates each were performed (represented as individual dots). Replicates with missing LC–MS/MS values were omitted from the plot. >2 independent experiments with missing values were reported as not reliably detected (ND) by proteomics analyses. Data represented as mean ± standard deviation. Statistical analysis was performed using Brown–Forsythe and Welch ANOVA or Kruskal–Wallis ANOVA by GraphPad software. *p < 0.05, **p < 0.01, ***p < 0.001, and ****p < 0.0001.

Efflux and influx transporters are well-characterized determinants of brain drug distribution. TFV and FTC are substrates of multiple influx and efflux transporters that may influence the heterogeneous ART distribution we observed in Figure 1. However, understanding of the differential expression of drug transporters among BBB cell types is limited, as most studies focused solely on endothelial cells.^29,30^ To address this gap, we evaluated TFV and FTC efflux transporters Multidrug Resistance Protein (MRP) 1 (MRP1) and MRP4, as well as FTC influx transporters Equilibrative nucleoside transporter 1 (ENT1), Organic Anion Transporter (OAT) 1 (OAT1), and OAT3, using proteomics by LC–MS/MS in primary human BBB cell monocultures. Our studies also included primary human hepatocytes as a comparative control as their transporter expression is well characterized (Figure 2). Protein expression was also evaluated by Western blotting (Figure 2).

We identified differential concentrations of ENT1 and MRP1 among the BBB cell types (Figure 2A,C), while MRP4 concentrations were consistent (Figure 2B). Endothelial cells had significantly higher ENT1 concentrations (239.9 ± 62.36 nM) relative to pericytes (143.0 ± 52.64 nM, *p* = 0.0136) and astrocytes (103.3 ± 62.88 nM, *p* = 0.0019) (Figure 2A). However, pericyte MRP1 concentrations (34.22 ± 6.90 nM) were 42% higher compared to astrocytes (23.99 ± 5.65, *p* = 0.0126) while remaining comparable to endothelial cells (29.48 ± 9.90 nM, *p* = 0.6060) (Figure 2C). We did not detect TFV influx transporters, OAT1 and OAT3, by LC–MS/MS-based proteomics; however, we previously showed their expression in endothelial cells.^16^ Western blot confirmed transporter expression in all of the BBB cells. While we did not reliably quantify ENT1, MRP4, and MRP1 in fresh primary human hepatocytes by proteomics (Figure 2A–C), it is accepted that they are variably expressed in this cell type, likely explaining our inability to reliably quantify these transporters by mass spectrometry-based proteomics.^31−33^ Nevertheless, we demonstrated that BBB cells differentially expressed several TFV and FTC efflux and influx transporters, which may explain their differential intracellular TFV and FTC accumulation.^16,24,25^

Next, we evaluated the nucleotide-metabolizing enzymes that facilitate TFV and FTC metabolism to TFV-DP and FTC-TP. Adenylate kinase 2 (AK2), Brain-type creatine kinase (CKB), Pyruvate kinase muscle (PKM), and Pyruvate kinase (PK), liver, and red blood cell (PKLR) were differentially quantifiable in BBB cells (Figure 2D–G). We determined that astrocytes (1626 ± 372.90 nM) expressed CKB approximately 3663% higher relative to endothelial cells (44.39 ± 18.03 nM, *p* < 0.0001) and 2191% higher than pericytes (74.24 ± 14.69 nM, *p* < 0.0001), supporting previous findings (Figure 2E).^34^ Additionally, CKB concentrations were over 67% higher in pericytes (74.24 ± 14.69 nM) relative to endothelial cells (44.39 ± 18.03 nM, *p* = 0.0066) (Figure 2E). PKM concentrations were over 224% higher in pericytes (5560 ± 1095 nM) relative to endothelial cells (2482 ± 1500 nM, p = 0.0022) (Figure 2G). As expected, CKM was not reliably detected in BBB cell types as its expression is limited in the brain (data not shown).^10^ Hepatocytes had minimal CKB and PKM concentrations relative to those of all BBB cell types (Figure 2E,G).

Similar differential concentrations of FTC-metabolizing enzymes also occurred in the BBB cells (Figure 2H–K). Pericytes had greater than 71% Deoxycytidine kinase (DCK) concentrations (232.1 ± 87.03 nM) relative to astrocytes (135.0 ± 40.64 nM, *p* = 0.0160) and endothelial cells (132.3 ± 52.88 nM, *p* = 0.0348) (Figure 2I). Interestingly, Phosphoglycerate kinase (PGK1) was most highly expressed in astrocytes (3951 ± 1569 nM), which had 223% higher concentrations compared to endothelial cells (1771 ± 1085 nM, *p* = 0.0391) (Figure 2J). While DCK and Thymidine kinase (TK1) were not reliably detected by LC–MS/MS-based proteomics in hepatocytes, these cells had the highest Cytidine/uridine monophosphate kinase 1 (CMPK1) and PGK1 concentrations relative to BBB cells, supporting their known importance in liver glycolysis (Figure 2H–K).^35,36^ Western blot confirmed BBB cell nucleotide-metabolizing enzyme expression. Together, we determined that pericyte and astrocyte TFV and FTC nucleotide-metabolizing enzymes are comparable to or significantly higher than endothelial cells. For many nucleotide-metabolizing enzymes, BBB cell concentrations were comparable to, or higher, than hepatocytes, indicating that these cells hold competitive ART-metabolizing capabilities as the liver. This was intriguing, as the brain is not typically regarded as a drug-metabolizing organ. These results highlight that BBB cells harbor the biotransformation machinery necessary to differentially metabolize TFV and FTC, identifying it as a pharmacologically relevant microenvironment wherein drug metabolism is a pertinent mechanism in determining CNS drug availability.

### TFV and FTC Transporters Are Differentially Active among BBB Cells

**Figure 3:**
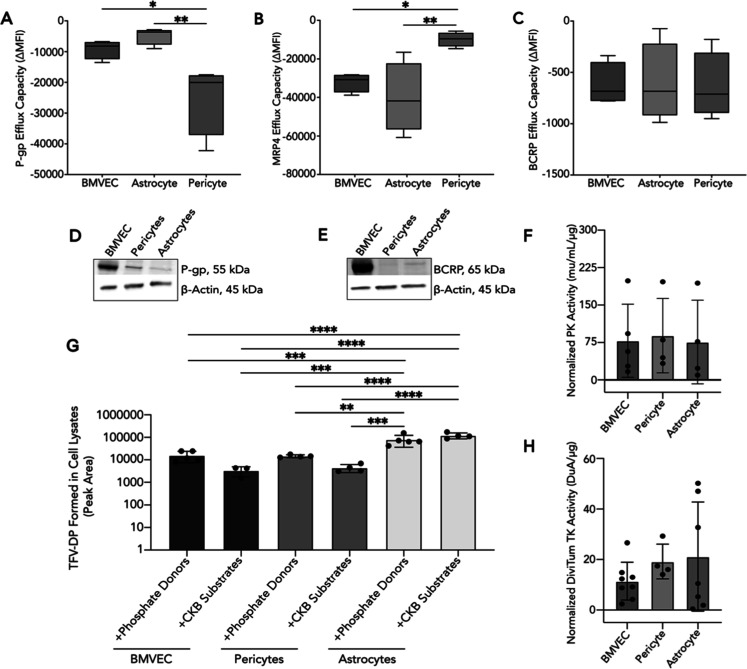
TFV/FTC efflux transporters and nucleotide-metabolizing kinases are differentially active in BBB cells. Brain microvascular endothelial cell, pericyte, and astrocyte monocultures were loaded with dyes for (A) P-gp (rhodamine 123, 10 μM), (B) MRP4 (monobromobimane, 10 μM), and (C) BCRP (Hoechst 33342, 5 μg/mL) for 15 min at 37 °C, 5% CO_2_. The dyes were allowed to efflux out for 2 h at 37 °C and 5% CO_2_, after which flow cytometry was performed to quantify intracellular fluorescence (mean fluorescence intensity, MFI) as an indicator of efflux capacity. Statistical analysis was performed using one-way ANOVA. (D) P-gp and (E) BCRP expressions were confirmed by Western blot, as these proteins were not reliably measurable by proteomics analyses. (F) Endogenous PK and (H) TK activities were measured in primary human BBB cell monocultures by a colorimetric activity assay and DiviTum activity assay, respectively. Four to eight independent experiments (represented by individual dots) were performed. Statistical analysis was performed using a Brown–Forsythe and Welch ANOVA test. (G) TFV-DP formation in human primary BBB cell lysates was measured by a CKB-mediated TFV metabolism assay using LC–MS/MS. Cell lysates were incubated with TFV-monophosphate (TFV-MP) and phosphocreatine (+CKB Substrates) or a mixture of phosphocreatine, phosphoenolpyruvate, and ATP (+Phosphate Donors) for 30 min at 37 °C. The reaction was quenched by LC–MS-grade methanol, and peak area of TFV-DP was measured by LC–MS/MS. Three to five independent experiments were performed (represented by individual dots). Statistical analysis was performed by using one-way ANOVA by GraphPad software. *p < 0.05, **p < 0.01, ***p < 0.001, and ****p < 0.0001.

Intrigued by the implications of the differential expression of TFV and FTC transporters and nucleotide-metabolizing enzymes, we evaluated whether this translated to distinct functional activity. Substrates without overlapping specificity do not exist for ENT1, MRP4, MRP1, OAT1, and OAT3, making it challenging to evaluate their differential activity in our primary BBB cells. Therefore, we evaluated the activity of additional efflux transporters known to interact with ART: Breast cancer resistance protein (BCRP), MRP4, and P-glycoprotein (P-gp) (Figure 3). Importantly, while we did not reliably quantify BCRP and P-gp in our BBB cell types with proteomics by LC–MS/MS, their expression was confirmed with Western blot (Figure 3D,E).

To evaluate efflux transporter activity, primary human BBB cell monocultures were loaded with a fluorescent dye (monobromobimane for MRP4, Hoechst 33342 for BCRP, and rhodamine 123 for P-gp) whose efflux is known to be mediated by our transporters of interest according to our established method.^16^ We then measured the remaining intracellular fluorescence after a sufficient time passed to facilitate dye efflux. Importantly, the fluorescent dyes were not toxic to the BBB cell types (Figure S2C,I,O), and all dye rapidly entered the viable cells, as indicated by near 100% positivity for Hoechst 33342, rhodamine 123, and monobromobimane (Figure S2D–F,J–L,P,R). Efflux of all dyes occurred within two h, denoted by the significant loss of intracellular fluorescence (Figure S3, black histograms compared to gray histograms).

We quantified the efflux capacity of each transporter by subtracting the difference in intracellular fluorescence, as indicated by the mean fluorescence intensity, following dye uptake and efflux (Figure S4). We determined that P-gp and MRP4 efflux capacities differed across cell types (Figure 3A,B). Pericytes had the highest P-gp efflux capacity (−24,671 ± 11,643) as it was 546% and 278% higher than astrocytes (−4520 ± 2823, *p* = 0.0080) and endothelial cells (−8879 ± 3060, p = 0.0296), respectively (Figure 3A). In contrast, pericytes had the lowest MRP4 efflux capacity (−9815 ± 3722) as it was 75% lower compared to astrocytes (−40,235 ± 18,231, *p* = 0.0095) and 69% lower than endothelial cells (−32,143 ± 4880, *p* = 0.0462) (Figure 3B). P-gp and MRP4 efflux activities were comparable between endothelial cells and astrocytes. Surprisingly, BCRP was the only efflux transporter for whom all BBB cells had comparable efflux activity (Figure 3C). It is important to note the discordance between efflux expression and activity among the BBB cells, demonstrating the importance of evaluating the transporter function. Our work determined that, while present, each BBB cell type expressed differentially active TFV and FTC transporters, which may explain cell-based differences in intracellular TFV and FTC accumulation.^16,24,25^ These findings demonstrate the importance of evaluating drug transport at the BBB, rather than viewing it as an extension of the liver, as it possesses distinct mechanisms that may contribute to differential drug disposition in the CNS. Additionally, our results elucidate the importance of considering pericytes and astrocytes, in addition to brain endothelial cells, in establishing transport function at the BBB.

### Astrocytes Have the Highest CKB Activity, while TK and PK Are Equally Active among All BBB Cell Types

We first evaluated CKB activity through our TFV activation activity assay, which has been previously used to measure CKB activity in tissues.^10^ In this assay, protein lysates from BBB cell monocultures were incubated for 30 min with the TFV-DP precursor, TFV-MP, together with phosphocreatine that served as a phosphate donor for the CKB-mediated conversion of TFV-MP to TFV-DP. To measure the activity of all TFV-MP metabolizing enzymes, the (1) PKM phosphate donor, phosphoenolpyruvate, (2) universal phosphate donor, ATP, and (3) phosphocreatine were incubated with protein from BBB monoculture lysates and TFV-MP. Peak areas of TFV-DP formed from the enzyme-catalyzed reactions were measured in each BBB cell type for the CKB-mediated conversion (+CKB substrate condition) and general TFV-MP conversion (+Phosphate Donors) (Figure 3G). TFV-DP formation in the CKB-mediated reaction was over 3500% higher in astrocytes (120,921 ± 35714) as compared to endothelial cells (3345 ± 1576, *p* = <0.0001) and pericytes (4439 ± 1744, *p* = <0.0001) (Figure 3G). This also occurred for TFV-DP formation in the general TFV-MP conversion, which was approximately 500% higher in astrocytes (79,229 ± 43,244) relative to endothelial cells (15,616 ± 8159, *p* = 0.0009) and pericytes (14,522 ± 2103, *p* = 0.0016) (Figure 3G). Of importance, no significant difference occurred in the TFV-DP peak area between the CKB-mediated reactions and the general TFV-MP conversion for all cell types, suggesting that CKB may be the primary enzyme to metabolize TFV-MP to TFV-DP at the BBB as no significant increase in TFV-DP occurred when adding reaction substrates for other TFV-activating enzymes. Minimal background from TFV-DP formation was observed in reactions where protein from cell lysate was incubated with TFV-MP without any phosphate donor added, demonstrating assay specificity (data not shown). Further, TFV-DP formation was not detected in negative controls that excluded assay substrates such as TFV-MP or BBB cell lysates (data not shown).

We next evaluated additional protein kinase activities. We measured endogenous PK function, where activity was determined by pyruvate and ATP formation from the PK-catalyzed reaction of phosphoenolpyruvate and ADP. PK activity was comparable across endothelial cells, pericytes, and astrocytes, though enzyme activity varied across experimental replicates (Figure 3H). Next, we investigated the function of the FTC-metabolizing enzyme, TK, using a DiviTum ELISA assay. TK endogenous activity was comparable among all BBB cell types (Figure 3H). However, similar to PK activity, significant variability occurred between measurements, particularly within astrocytes (Figure 3F). Our findings demonstrate that while endogenous PK and TK activities are comparably active among BBB cells, CKB involvement in catalyzing TFV-DP formation occurs in a cell-type-specific fashion (Figure 3F,H,G).

### HIV and ART Regulate TFV and FTC Transporters and Nucleotide-Metabolizing Enzymes in Astrocytes and Pericytes but Not in Endothelial Cells

**Figure 4:**
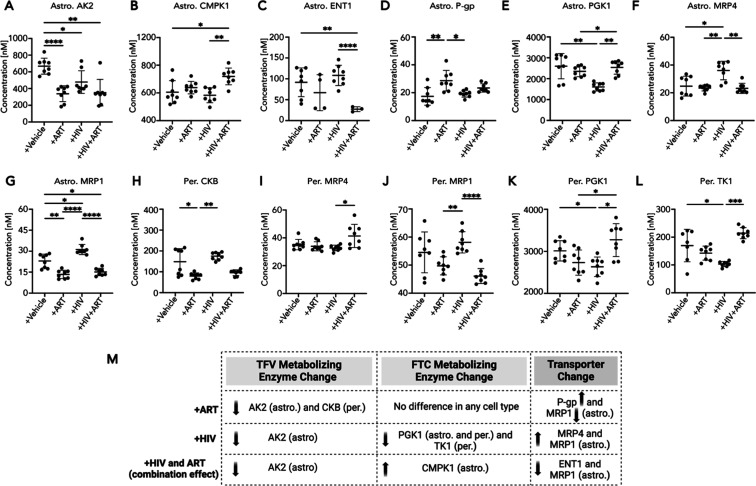
BBB TFV/FTC nucleotide-metabolizing kinases and transporters are impacted in a cell-dependent manner by ART and HIV. Primary human pericyte and astrocyte monocultures were exposed to ART (10 μM TFV, 10 μM FTC, and 10 μM DTG), HIV (5 ng/mL), or HIV + ART (10 μM TFV, 10 μM FTC, 10 μM DTG, and 5 ng/mL HIV) for 24 h at 37 °C, 5% CO_2_. Treatment with a vehicle was used as a control. Pericytes and astrocytes were lysed and processed for proteomics analyses. Concentrations of proteins of interest were measured by Proteomic Ruler. After exposure, changes in protein concentration were assessed in (A–G) astrocytes (astro) for (A) AK2, (B) CMPK1, (C) ENT1, (D) P-gp, (E) PGK1, (F) MRP4, and (G) MRP1. Similarly, changes in protein concentration were assessed in (H–L) pericytes (per) for (H) CKB, (I) MRP4, (J) MRP1, (K) PGK1, and (L) TK1. The significant changes (A–L) in the concentrations of TFV and FTC transporters and metabolizing enzymes after ART, HIV, or HIV + ART exposure relative to vehicle were (M) summarized for astrocytes (astro) and pericytes (per). An arrow next to each protein denotes an increase (upward arrow) or decrease (downward arrow) in protein concentration after ART, HIV, or HIV + ART exposure relative to the vehicle. Four independent experiments with two LC–MS/MS injection replicates each were performed per condition (represented as individual dots). Replicates with missing LC–MS/MS values were omitted from the plot. >2 independent experiments with missing values were reported as not reliably detected (ND) by proteomics analyses. Data represented as mean ± standard deviation. Statistical analysis was performed using Brown–Forsythe and Welch ANOVA or Kruskal–Wallis ANOVA by GraphPad software. *p < 0.05, **p < 0.01, ***p < 0.001, and ****p < 0.0001.

There is a substantial impact of HIV, viral proteins, and ART on the BBB; however, most studies focused primarily on BBB permeability and integrity while not considering the components involved in ART transport and metabolism.^37,38^ To evaluate these processes more comprehensively at the BBB in a condition more physiologically relevant to people living with HIV, we evaluated whether ART or HIV regulated TFV and FTC transporters and nucleotide-metabolizing enzymes. To accomplish this, we treated our primary human BBB cell monocultures with ART, HIV, combined HIV and ART, or vehicle for 24 h and performed LC–MS/MS-based proteomics. We used HIV and ART concentrations previously measured in vivo and commonly used in *in vitro* experiments (Figure 4).^25^

We found that the concentrations of several TFV and FTC transporters and nucleotide-metabolizing enzymes were significantly impacted by 24 h treatment. Strikingly, these changes occurred only in astrocytes and pericytes but not endothelial cells. HIV, ART, and combined HIV and ART treatment created distinct clustering of pericyte and astrocyte proteomes (Figure S5). However, this did not occur for endothelial cells, as they did not form separate clusters (Figure S5). This biased impact of HIV and ART was further confirmed as significant changes in TFV and FTC transporter and nucleotide-metabolizing enzyme concentrations occurred only in pericytes and astrocytes (Figure 4) but not in endothelial cells (Figure S6).

We therefore focused our efforts on understanding the impact of ART and/or HIV on pericytes and astrocytes. ART treatment significantly decreased the concentration of AK2 (338.30 ± 94.74 nM, *p* = <0.0001) and MRP1 (13.38 ± 2.96 nM, *p* = 0.0028) by ∼50% in astrocytes, as well as CKB (160.30 ± 15.04 nM, *p* = 0.0335) by 30% in pericytes (Figure 4A,G,H). In contrast, ART increased P-gp concentrations by 65% in astrocytes (28.60 ± 7.35 nM, *p* = 0.0069) (Figure 4D). Next, we examined how HIV altered our transporters and metabolizing enzymes of interest. After HIV exposure, TFV and FTC nucleotide-metabolizing enzyme concentrations significantly decreased for AK2 (477.70 ± 135.60 nM, *p* = 0.0356) and PGK1 (1522 ± 194.20 nM, *p* = 0.0032) by ∼30% in astrocytes (Figure 4A,E). In pericytes, HIV decreased PGK1 (2632 ± 233.50 nM, *p* = 0.0356) and TK1 (103.40 ± 9.30 nM, *p* = 0.0409) by 13% and 40%, respectively (Figure 4K,L). Interestingly, TFV and FTC transporter concentrations increased after HIV exposure for MRP4 (36.02 ± 6.63 nM, *p* = 0.0314) by 45% and by 35% for MRP1 (31.22 ± 3.61 nM, *p* = 0.0117) in astrocytes, but there were no impacts on TFV/FTC transporter concentrations in pericytes (Figure 4F,G,I,J).

Finally, we determined the combined impact of HIV and ART. Combined HIV and ART treatment decreased AK2 and MRP1 concentrations relative to vehicle concentrations only for astrocytes but not pericytes (Figure 4A,G). Interestingly, astrocyte exposure to the combination of HIV and ART resulted in a synergistic effect for CMPK1 and ENT1, where the concentrations of these proteins changed only upon dual treatment, not with their individual exposure (Figure 4B,C). Astrocyte CMPK1 concentrations increased by 18% (719.0 ± 60.48 nM, *p* = 0.0448), while ENT1 concentrations decreased by 70% (27.41 ± 5.89 nM, *p* = 0.0050) (Figure 4B,C). In contrast, neither of these proteins were changed in pericytes with any treatment condition (data not shown). The changes in astrocyte and pericyte transporter and metabolizing enzyme concentrations after ART, HIV, and a combination of HIV and ART exposure were summarized (Figure 4M). Taken together, these data indicate that ART, HIV, and combination treatment may profoundly impact the transport and metabolic capacity of the BBB, which occurs by regulating pericytes and astrocytes but not endothelial cells and may result in differential intracellular levels of ART and treatment of HIV in the CNS (Figure S6). Consequently, differentially regulated drug transport and metabolism at baseline and in disease may impact heterogeneous CNS drug distribution and efficacy. Further, there is a protein-specific manner for which exposure to HIV, ART, or a combination of HIV and ART selectively regulates proteins involved in TFV and FTC transport and metabolism.

### ART and HIV Modulate Astrocyte and Pericyte Cell Transport and Regulation, Metabolism, Immune Response, and Cell–Cell Communication Pathways

**Figure 5:**
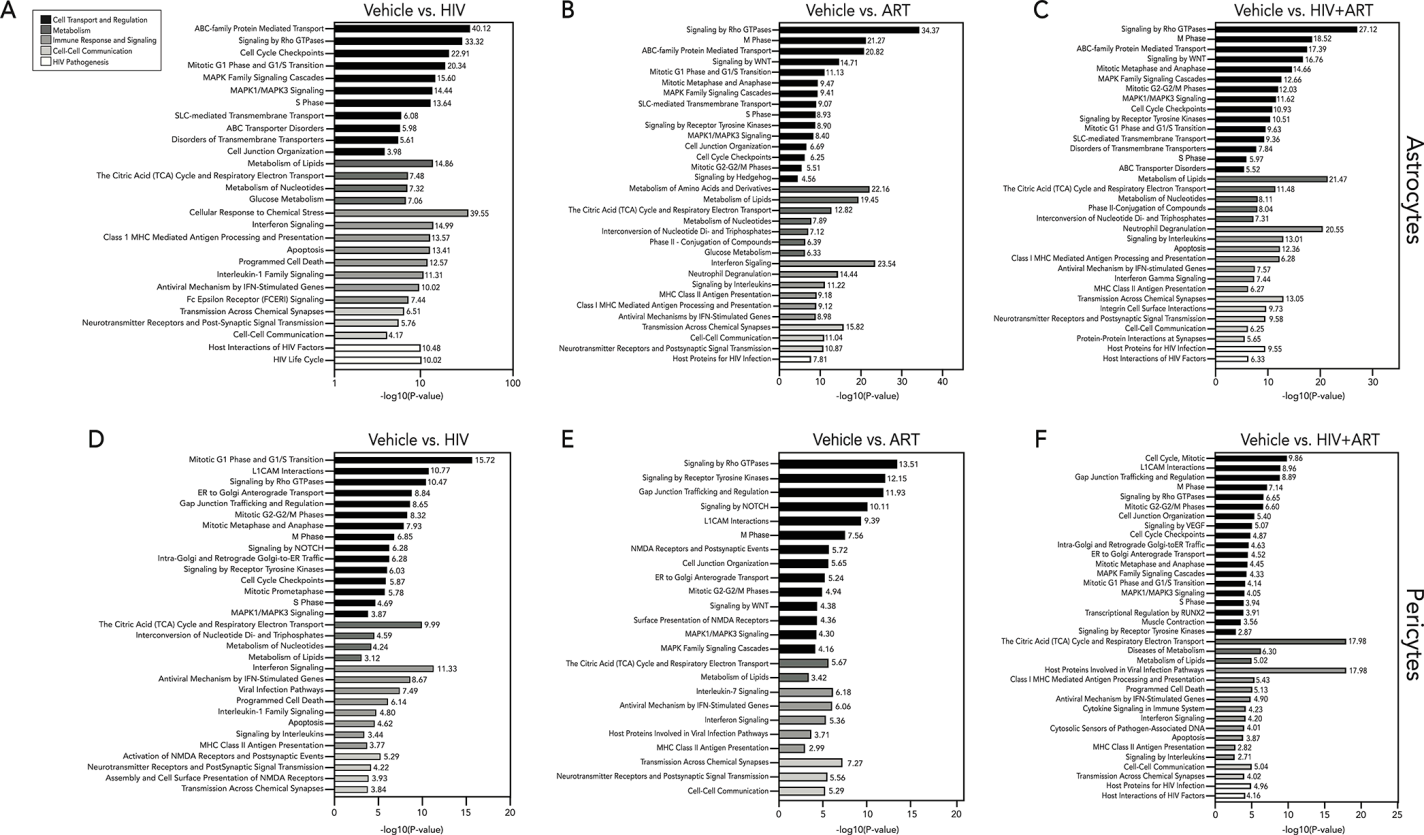
HIV and ART differentially impact astrocyte and pericyte cell pathways involved in cell transport and regulation, metabolism, immune response and signaling, and cell–cell communication. Primary human pericyte and astrocyte monocultures were exposed to ART (10 μM TFV, 10 μM FTC, and 10 μM DTG), HIV (5 ng/mL), or HIV + ART (10 μM TFV, 10 μM FTC, 10 μM DTG, and 5 ng/mL HIV) for 24 h at 37 °C and 5% CO_2_. Treatment with the vehicle was used as a control. Pericytes and astrocytes were lysed and processed for proteomics analyses. Pathways significantly altered in astrocytes after (A) HIV, (B) ART, and (C) HIV + ART exposure as well as in pericytes after (D) HIV, (E) ART, and (F) HIV + ART (relative to the vehicle) were analyzed by SimpliFi proteomics software. Pathways of interest were filtered by pathway changes greater than one log fold and hypergeometric *p*-value <0.05. Pathway hits of interest were ranked by –log10 (hypergeometric p-value) calculated by SimpliFi statistics. Pathway analyses reflect four independent experiments per condition with two LC–MS/MS injection replicates each.

While we focused primarily on TFV and FTC transporters and metabolizing enzymes, we also recognize that HIV and ART impact additional cellular functions at the BBB as the BBB responds dynamically to disease that may lead to dysregulation—thereby exacerbating neurologic injury and dysfunction—and may influence ART CNS availability. So, we next evaluated pharmacologically related cellular pathways that may contribute to the transporter and metabolizing enzyme concentration changes we observed (Figure 4) following HIV, ART, and combination treatment. We used SimpliFi to accomplish this by further processing and analyzing the BBB proteomes to identify additional cellular pathways of interest. We identified several proteins as significantly impacted by HIV or ART exposure (*p* < 0.05), relative to the vehicle. To summarize and organize these findings, we further filtered differentially expressed proteins such that the log2 fold change within affected pathways of interest relative to the vehicle was greater than 0. We found that the resulting top hits in both astrocytes and pericytes (*p* < 0.05, log2 fold change >0) were pathways involved in (1) drug transport and cell regulation, (2) drug metabolism, (3) immune response and signaling, (4) cell–cell communication, and (5) HIV pathogenesis (Figure 5). Astrocytes are actively infected by HIV, and thus, we were not surprised to identify several pathways implicated in immune responses and signaling and HIV pathogenesis in HIV-exposed astrocytes (Figure 5A,C).^27^ Pericytes are also permissive to HIV, and we observed pathway hits involved in host interactions for HIV pathogenesis and immune response and signaling after exposure to HIV, ART, and a combination of HIV and ART, which were characterized by a mixture of proteins that were both upregulated and downregulated (Figure 5D–F).^39^ Pathway analysis of astrocytes and pericytes identified significant pathway hits involved in ART metabolism and transport, as well as glucose metabolism, cell regulation, cell cycle, and cell signaling after exposure to HIV, ART, and combination treatment. These pathway hits may contribute to the changes we previously observed (Figure 4) in transporter and metabolizing enzyme concentrations after HIV and ART exposure as these same proteins are involved in many of the affected pathways (Figure 5). The significant changes in BBB cell pathways involved in drug metabolism and transport after exposure to HIV, ART, and combination treatment may further impact the CNS drug disposition in the context of disease. This highlights the importance of further investigating these pathways that may contribute to the dynamic pharmacological nature of BBB cells (Figure 5). Similar to that which occurred for TFV and FTC transport and metabolizing proteins, endothelial cell exposure to HIV, ART, and combined HIV and ART treatment had minimal impact in these pathways (Figures S6K,L,M). This was unexpected as previous studies reported changes in brain endothelial cell pathways, such as transport, after exposure to ART or the HIV protein, tat.^38,40,41^ The disparity in our findings may occur due to our use of clinically relevant concentrations of ART and using replication competent HIV, rather than a viral protein. We propose that evaluation of CNS drug disposition occurs not only in baseline conditions but also in the diseased state, as the fundamental physiology of the BBB, including drug transporters and metabolizing enzymes, differs during pathology, effectively shaping CNS drug availability.

In summary, first-line ART drugs, TFV and FTC, were evaluated as representative models of CNS-penetrant small-molecule drugs with significant disease relevance to determine mechanisms at the BBB that may govern heterogeneous CNS drug disposition. We establish a foundation regarding BBB cells as pharmacological entities that form the dynamic BBB microenvironment and may determine CNS drug access, as they possess machinery to differentially transport and metabolize CNS-penetrant drugs. Our findings provide evidence of dynamic pharmacological capabilities of metabolism and transport within BBB cells and provide further considerations of region-specific differences in CNS drug metabolism, transport, and distribution. Additionally, our results highlight that mechanisms of drug transport and metabolism at the BBB may be impacted by therapeutic intervention and disease, which may influence the CNS drug disposition.

## Methods

### Cell Lines

Primary human astrocytes (ScienCell Research Laboratories, Carlsbad, CA) and brain microvascular endothelial cells were cultured as described previously.^16^ Astrocytes were used at passages 1–6 for all experiments, and endothelial cells were used at passages 2–12 for all experiments. Information on donor including sex and age was not available from source.

Primary human pericytes (ScienCell Research Laboratories) were grown to confluence on 1.5% poly-l-lysine-coated flasks (Sigma, St. Louis, MO) in Pericyte Medium (ScienCell Research Laboratories), containing HEPES and sodium bicarbonate buffered to pH 7.4. Media were supplemented with 2% fetal bovine serum (FBS) (ScienCell Research Laboratories), 1% penicillin–streptomycin 10,000 U/mL (Gibco, Grand Island, NY), and 1% pericyte growth supplement (ScienCell Research Laboratories). Pericytes were used at passages 2–13 for all experiments. Information on donor including sex and age was not available from source.

Fresh primary human hepatocytes (BioIVT, Westbury, NY) were purchased in a liquid suspension and isolated from a deceased Caucasian biological female, age 66 with a healthy medical background.

### Animals

Four male and three female rhesus macaques (*Macaca mulatta*), all four years old, were included in this study. Rhesus macaques were selected by prescreening negative for the immunoprotective MHC class I alleles Mamu-A*01, Mamu-B*08, and Mamu-B*17. All rhesus macaques were approximately 6 kg throughout the study and when tissues were harvested. All protocols were performed at The Johns Hopkins University School of Medicine and Emory University School of Medicine in compliance with the guidelines of the Animal Care and Use Committees, the US Department of Agriculture Animal Welfare Act, and the NIH Guide for the Care and Use of Laboratory Animals.

### Rhesus Macaque Experimental Design

We used a well-established rhesus macaque (*Macaca mulatta*) model^7,14,42^ using samples from two cohorts. The first cohort involved two SIV-infected, ART-treated rhesus macaques (M1 and M2) and one healthy, ART untreated rhesus macaque (M3) that were inoculated intravenously using SIVmac251 as previously described.^43^ At day 42 post infection, a subcutaneous dose of 2.5 mg/kg dolutegravir (DTG, ViiV, London, England, UK), 20 mg/kg TFV (Gilead, Foster City, CA), and 40 mg/kg FTC (Gilead) was administered once daily for six months. Euthanasia occurred at approximately 200 days postinoculation. The second cohort involved five uninfected, ART-treated rhesus macaques (M4–M5, F1–F3). ART administration and euthanasia occurred in the same manner as described for cohort one animals, except an intramuscular dose of 2.5 mg/kg dolutegravir (DTG, ViiV), 5.1 mg/kg TFV (Gilead), and 50 mg/kg FTC (Gilead) was used.

### Primary Rhesus Macaque Astrocyte Isolation

To isolate rhesus macaque astrocytes, blood vessels were surgically removed from three female and two male rhesus macaque whole brain sections, washed with 1× phosphate buffered saline (Gibco), and digested for 30 min at 37 °C, 5% CO_2_ with a trypsin solution containing 10% of 2.5% trypsin (Thermo Fisher Scientific, Waltham, MA). Digested brain tissue was filtered twice using 183 μm sterile mesh (Corning, Corning NY) and a 100 μm nylon cell strainer (Corning) in 10% DMEM (Thermo Fisher Scientific). The resulting brain capillaries were removed from the 100 μm nylon cell strainer (Corning) and lysed in 70% LC–MS-grade methanol for mass spectrometry analyses. Astrocytes were then isolated using a density gradient containing Percoll (Thermo Fisher Scientific), filtered through a 40 μm nylon cell strainer (Corning), and washed with DMEM-10 supplemented with 15% FBS (Thermo Fisher Scientific), 0.1% Geneticin (Thermo Fisher Scientific), and 100 mM sodium pyruvate. Isolated astrocytes were lysed in 70% LC–MS-grade methanol. Proteomics by mass spectrometry was used to authenticate isolated astrocytes.

### Immunofluorescent Microscopy

Primary brain endothelial cells, pericytes, and astrocytes were seeded (1.6 × 10^4^ cells/dish) on 35 mm ibiTreat dishes (Ibidi USA, Madison, WI). ibiTreat dishes were coated with 0.2% gelatin and 1.5% poly-l-lysine prior to seeding brain endothelial cells and pericytes, respectively. Cells were fixed, stained, and imaged as described previously.^16^ Cells were probed with antibodies with specificity to GFAP (53-9892-82, Invitrogen, Carlsbad, CA), VE-Cadherin (14-1449-82, Invitrogen), or ANPEP (MA5-32226, Invitrogen) overnight at 4 °C, washed three times with DPBS at room temperature, and probed with the appropriate Alexa Fluor 488 conjugated secondary antibody (ab97051 and A-11001, Abcam, Cambridge, MA) for 1 h at room temperature. 10–20 representative images were acquired for each cell type 1–15 days postfixation using the Echo Revolution (San Diego, CA) in inverted mode with a 20× Plan Apo objective, with 0.8 numerical aperture. Channels used included blue for cell nuclei, red for cell morphology, and green for proteins of interest. Prior to image acquisition, the focal plane on the *Z*-axis was determined manually, and exposure time and light intensity were adjusted to optimize signal-to-noise ratio and avoid saturation.

### ART Intracellular Concentration Determination

Upon reaching 90% confluency, endothelial cells, pericytes, and astrocytes were treated with TFV (18 μM), FTC (2 μM), DTG (6 μM), or vehicle, for 24 h at 37 °C, 5% CO_2_ (TFV, FTC, and DTG were all from Toronto Research Chemicals, Toronto, Canada). TFV and FTC were prepared in a 100 mM stock solution in sterile water, while DTG was diluted in DMSO together with sterile water. All subsequent dilutions of DTG were made in sterile water, and the final concentration of DMSO was less than 0.1%. Of note, ART concentrations reflect the approximate equivalent concentrations of the macaque ART dosing regimens used in this study.

In vitro ART-treated brain endothelial cells, pericytes, and astrocytes as well as rhesus macaque brain tissue and isolated astrocytes were lysed in 70% LC-MS-grade methanol. FTC, TFV, FTC-TP, and TFV-DP were quantified in collaboration with the Clinical Pharmacology Analytic Laboratory at The Johns Hopkins University School of Medicine as previously described.^44,45^ Each experimental replicate of endothelial cells, pericytes, and astrocytes includes data collected from three to four independent experiments. Experimental replicates for astrocytes isolated from rhesus macaques were collected from five independent animals. Lastly, each experimental replicate for ART-treated rhesus macaque brain includes data collected from two independent animals.

### Western Blot

Endothelial cells, pericytes, and astrocytes were lysed with 1× RIPA buffer (Cell Signaling Technology, Danvers, MA) supplemented with a 1× protease/phosphatase inhibitor (Cell Signaling Technology). Total protein concentrations were determined by Bradford Assay with the Bio-Rad Protein Assay Dye reagent concentrate (Bio-Rad, Hercules, CA) following the manufacturer’s instructions. Western blots were performed as previously described.^16^ Blots were probed with antibodies with specificity to BCRP (NBP2-22124, Novus Bio, Centennial, CO), P-gp (PA5-61300, Invitrogen), MRP4 (12705, Cell Signaling Technology), MRP1 (Ab24102, Abcam), ENT1 (PA5-116451, Invitrogen), PGK1 (PA5-28612, Invitrogen), AK2 (11014-1-AP, Proteintech, Rosemont, IL), CKB/CKM (15137-1-AP, Proteintech), TK (15691-1-AP, Proteintech), CMPK (11360-1-AP, Proteintech), DCK (17758-1-AP, Proteintech), PKM1 (15821-1-AP, Proteintech), and PKLR (2456-1-AP, Proteintech), overnight at 4 °C, washed with TBS-T, and probed with the appropriate secondary antibody (ab97023 and Ab97051, Abcam) for 1 h at room temperature. All antibodies were used at concentrations recommended by the manufacturer. Western Lightning Plus-ECL (PerkinElmer, Waltham, MA) was used as a chemiluminescence substrate, and the signal was detected with the Azure Biosystems c600 Imager (Azure Biosystems, Dublin, CA). As a loading control, membranes were stripped with Restore Plus Western Blot Stripping Buffer (Thermo Fisher Scientific) and reprobed with antibody against β-Actin HRP (8H10D10, Cell Signaling Technology) for 1 h at room temperature.

### BBB Monoculture Proteomics by LC–MS/MS

Endothelial cells, pericytes, and astrocytes were lysed with a 1× proteomics lysis buffer containing 5% sodium dodecyl sulfate (SDS) (Invitrogen) and 50 mM triethylammonium bicarbonate (Sigma). Cell lysates were processed for proteomics by mass spectrometry and analyzed as previously described^10^ with the exception that proteins were digested into peptides using the S-Trap micro spin columns (ProtiFi, Fairport, NY) and desalted using the Pierce peptide desalting spin columns (Thermo Fisher Scientific) according to the manufacturer’s instructions. Resulting spectra were uploaded to Spectronaut 18.7 (Biognosys, Cambridge, MA). Peptides were identified and quantified using the directDIA analysis default settings with the proteotypicity filter set to “Only Proteotypic” and a “methylthio” variable modification. MS1 protein group quantifications, UniProt numbers, and molecular weights from Spectronaut (Biognosys) were imported into Perseus (Max Planck Institute of Biochemistry, Planegg-Martinsried, Bavaria, Germany) for use of the Proteomic Ruler plug-in, as previously described.^46^ The default proteomic ruler plug-in settings were used for the hepatocytes. To account for differences in cell size, the total cellular protein concentration for endothelial cells, pericytes, and astrocytes was set to 100 g/L as a conservative estimate according to previous studies.^47^ The scaling mode and ploidy were set to the default proteomic ruler plug-in settings. Protein concentrations (nM) and copy numbers, as estimated by the Proteomic Ruler, were used for analysis. Proteins were reported as “not reliably quantified or detected (ND)” by proteomics when one or less unique peptide was identified for the corresponding protein. Protein relative abundances and Proteomic Ruler concentrations and copy numbers of all identified proteins were made publicly available in The Human Blood Brain Barrier Monoculture Proteome Repository 1.0.2 (https://hannahwilkins.shinyapps.io/Blood_Brain_Barrier_Cells_Proteomics_Repository_Publish/). Proteomics raw data can be directly accessed at massive.ucsd.edu as data set MSV000094702 using the password: WilkinsBBB.

### In Vitro CKB-Mediated TFV Enzyme Activity Assay

Primary human endothelial cells, pericytes, and astrocytes were lysed at 100% confluency with an assay reaction buffer containing 75 mM HEPES pH 7.5 (Gibco), 5 mM MgCl_2_ (Thermo Fisher Scientific), 50 mM KCl (Sigma), 2 mM dithioerythritol (Sigma), and LC–MS-grade H_2_O (Thermo Fisher Scientific). Activity assay reactions were performed as previously described.^10^ Control reactions were performed in parallel by omitting the addition of initiation buffer, cell lysate, or TFV-MP. Independent experimental assay reactions were completed for endothelial cells (*n* = 3–5), pericytes (*n* = 4), and astrocytes (*n* = 4–5).

TFV-DP formation was measured by triple quadrupole LC–MS/MS as previously described,^10^ with the exception that samples were analyzed on an Agilent Ultivo Triple Quadrupole LC/MS (Agilent, Santa Clara, CA). Mobile-phase A was 5 mM *N*,*N*-dimethylhexylamine (DMHA) (Sigma) in water at pH 7.0, and mobile-phase B was 5 mM DMHA in 50% acetonitrile/50% water (v/v). Analytes were separated on a Halo C18 column (2.1 mm × 100 mm, 2.1 μm, Mac-Mod Analytical) (Mac-Mod Analytical, Chadds Ford, PA) at a flow rate of 0.450 mL/min using the following gradient: 5% B from 0 to 1 min, 5%–45% B from 1 to 9 min, 45% B from 9 to 13 min, 45%–100% B from 13 to 15 min, 100% B from 15 to 20 min, 100%–5% B from 20 to 23 min, and 5% B from 23 to 30 min. Analytes were ionized by heated electrospray ionization with a capillary voltage of 4800 V, gas temperature of 260 °C, gas flow rate of 11.0 L/min, and nebulizer pressure of 30 psi. Chromatographic peak areas were used for relative comparisons of the metabolite abundance.

### Efflux Transporter Activity Assay and Flow Cytometry

Efflux transporter activity was determined by measuring intracellular fluorescence following uptake of rhodamine 123 (10 μM, Thermo Fisher Scientific), Hoechst 33342 (5 μg/mL, Thermo Fisher Scientific), and monobromobimane (10 μM, Thermo Fisher Scientific), fluorescent substrates with specificity for P-gp, BCRP, and MRP4, respectively. Hoechst 33342 is a well-established transport substrate of BCRP; however, evidence exists that there is substrate specificity overlap with P-gp, which may contribute to mixed efflux effects.^48^ We did not observe intracellular trapping of monobromobimane or oxidative stress to induce cell death (data not shown).

Primary human brain endothelial cells, pericytes, and astrocytes were seeded onto six-well plates at 0.3 × 10^6^ cells/well. Upon reaching 100% confluency, cells were incubated with each fluorescent substrate for 15 min at 37 °C, 5% CO_2_ to allow uptake into the cell, after which fresh media were added, and the fluorescent substrates were allowed to efflux from the cells for 2 h at 37 °C, 5% CO_2_. Addition of the fluorescent substrate for 15 min at 37 °C and 5% CO_2_ that was not permitted to efflux was used as a positive control to determine maximal substrate uptake. Following exposure to the fluorescent substrates, cells were washed with PBS (Gibco), filtered using BD FACS tubes (BD Biosciences, Franklin Lakes, NJ) with cell strainer caps with 35 μm pores (BD Biosciences), and immediately subjected to flow cytometric acquisition where at least 10,000 singlet events were acquired with a BD LSRFortessa cytometer and Diva software version 9 on the Windows 10 platform (BD Biosciences). Flow cytometric data were analyzed using FlowJo version 10.9 (FlowJo, Ashland, OR) where fluorescence intensity of substrates in cells that received substrate for 2 h (efflux) was subtracted from the cells that received substrate at the end of the 2 h (uptake) to quantitate efflux capacity.

### Cell Viability Determination

Brain endothelial cells, pericytes, and astrocytes were exposed to rhodamine 123 (10 μM, Thermo Fisher Scientific), Hoechst 33342 (5 μg/mL, Thermo Fisher Scientific), and monobromobimane (10 μM, Thermo Fisher Scientific) for 15 min and 2 h, after which time viability was assessed, as previously described,^16^ using the BD Horizon Fixable Viability Stain 520 (BD Biosciences). Heat shock at 55 °C for 10 min served as a positive control for cell death. The cells were analyzed by flow cytometry within 5 min of staining.

### TK Activity Assay

When 100% confluent, endothelial cell, pericyte, and astrocyte monoculture cells were lysed with DiviTum TK activity lysis buffer (Biovica International, San Diego, CA). To determine TK activity, the DiviTum (Biovica International) TK activity assay was performed according to manufacturer’s instructions, as previously described.^49^ The absorbance readings to DiviTum units per liter (Du/L) were converted using the values from standards with known TK activity, with a working range from 20 to 4000 Du/L. TK activity was reported as the DiviTum TK activity value (DuA) per total protein (μg). The assays were performed at the Biovica laboratory in San Diego, California, and investigators were blinded to the cell type. TK activity was normalized to individual protein amount in brain endothelial cell, pericyte, and astrocyte lysates as measured by the Pierce Bicinchoninic acid (BCA) assay (Thermo Fisher Scientific).

### PK Activity Assay

PK activity was determined using the pyruvate kinase activity assay kit (Abcam) according to the manufacturer’s instructions. When 100% confluent, primary human brain endothelial cells, pericytes, and astrocytes were lysed using cold PK assay buffer, diluted, and analyzed by a colorimetric assay where the absorbance of the oxidized pyruvate was measured at 570 nm at 25 °C. Absorbance readings were taken by a Synergy HT microplate reader (BioTek, Winooski, VT) every minute for 20 min. PK activity was calculated using the concentration of pyruvate from the pyruvate standard curve generated in the assay over 20 min. PK activity was normalized to individual protein amounts in brain endothelial cell, pericyte, and astrocyte lysates as measured by the Pierce BCA assay (Thermo Fisher Scientific).

### BBB Cell HIV and ART Exposure

Upon reaching 90% confluency, primary human endothelial cells, pericytes, and astrocytes were washed with PBS (Gibco) and treated with ART, including TFV (Toronto Research Chemicals), FTC (Toronto Research Chemicals), DTG (Toronto Research Chemicals), HIV_ADA_ (5 ng/mL) (National Institutes of Health), ART in combination with HIV, or vehicle for 24 h at 37 °C, 5% CO_2_. All treatments were constituted in cell culture media. Final ART concentrations were 10 μM, which reflects the maximum serum concentrations (*C*_max_) of drug obtained from clinical studies.^24^ Following treatment, cells were washed twice with PBS (Gibco) and lysed in 1X proteomics lysis buffer, containing 5% SDS (Invitrogen) and 50 mM triethylammonium bicarbonate (Sigma). Cell lysates were processed for proteomics as described above.

## Supporting Information Available

Additional experimental data, including BBB cell monoculture microscopy, flow cytometry gating strategy, principal component analysis, and endothelial proteome data (PDF)

## Abbreviations

AK2: adenylate kinase 2
ANPEP: alanyl aminopeptidase
ARTs: antiretroviral therapies
BBB: blood brain barrier
BCRP: breast cancer resistance protein
CKB: brain-type creatine kinase
CMPK1: cytidine/uridine monophosphate kinase 1
CNS: central nervous system
CSF: cerebrospinal fluid
DCK: deoxycytidine kinase
DMHA: *N*,*N*-dimethylhexylamine
DTG: dolutegravir
ENT1: equilibrative nucleoside transporter 1
FTC: emtricitabine
FTC-TP: emtricitabine-triphosphate
GFAP: glial fibrillary acidic protein
HIV: human immunodeficiency virus
LC–MS/MS: liquid chromatography/mass spectrometry
MRP: multidrug resistance protein
MRP1: multidrug resistance protein 1
MRP4: multidrug resistance protein 4
OAT: organic anion transporter
OAT 1: organic anion transporter 1
OAT 3: organic anion transporter 3
PGK1: phosphoglycerate kinase
P-gp: P-glycoprotein
PK: pyruvate kinase
PKLR: pyruvate kinase, liver and red blood cell
PKM: pyruvate kinase muscle
SIV: simian immunodeficiency virus
TFV: tenofovir
TFV-MP: tenofovir-monophosphate
TFV-DP: tenofovir-diphosphate
TK1: thymidine kinase
